# The United Kingdom Hospitals Conference, 1908

**Published:** 1908-04-11

**Authors:** 


### HOSPITAL ADMINISTRATION.

### CONSTRUCTION AND ECONOMICS.

### THE UNITED KINGDOM HOSPITALS CONFERENCE, 1908.

The first meeting of the Hospital Conference of 1908 was held on the afternoon of April 1 in the Botanical Lecture Theatre of University College. After the Chairman of the Conference Committee (Mr. Charles Lupton, of the Leeds General Infirmary) had been unanimously elected to the chair of the meeting, and the transaction of formal business, Mr. H. C. Gosnell asked to be allowed to bring forward an amendment to the proposals of the last Conference, although he had not given notice of such an intention. Mr. Gosnell having explained that the rules requiring such notice had not reached the City of London Lying-in Hospital, which he had the honour to represent, the meeting consented to consider the amendment. The substance of the latter was concerned with the definition of the phrase "inability to pay" in No. 1 of the suggested model principles of hospital management adopted by the last Conference in December 1906. Mr. Gosnell holds very strongly that among the middle classes, and especially the lower middle classes, there are many who are commonly held to be able to pay for their own medical treatment because they have not the outside appearance of poverty. It is this class of persons whose obligations are so much in excess of their means that they are really worse off than the labouring classes, that Mr. Gosnell sought to bring clearly within the definition of those eligible for gratuitous treatment in hospitals. Respectability in dress should not be, as it too often is, regarded with suspicion by hospital authorities, if it can be shown that proper medical attention cannot be procured outside. It ought not to go abroad that hospitals exist only for the benefit of the working classes.

Mr. R. B. Dodson, of the Hospital for Women, Brighton, seconded the amendment, although he was not at all sure that Mr. Gosnell's aims would not be just as well secured by the resolution as it already stood. He instanced from his own experience many cases of distress amongst highly deserving lodging-house keepers, who are freely admitted to the benefits of the charity with which he is connected.

The Chairman said it was a question whether an explanation should be added to the recommendation of the committee. He thought the wording of the proposal and of the subsequent proposals rendered such explanation unnecessary.

Although the sympathy of the meeting was clearly with the object sought by the mover of the amendment, it was equally clear that the delegates were of opinion that the original resolution is in no way antagonistic to the objects sought to be achieved, and the amendment, when put to the vote, was lost by a large majority.

At this point a member of the Conference inquired of the Chairman, on behalf of the Board which he represented, as to the truth or otherwise of questions raised by the *Hospital Gazette* as to various alleged ulterior motives of those who had convened the Conference, and especially as to suggestions that manipulations in the interests of the medical profession were imminent.

The Chairman said that the names of the members of the Committee were alone sufficient reply to such allegations. He gave an unqualified denial to all such statements, pointing out that the great majority of those present were not medical men at all.

Mr. Harry Johnson, of the Leicester Infirmary, then moved the first resolution on the agenda paper. His motion was "That it is desirable to supply gratuitous treatment to those who are provided for by the Workmen's Compensation Act." After explaining exactly what the provisions of the Acts are, he said that the payments therein detailed are so meagre that the beneficiaries do not thereby become disqualified for hospital treatment, or liable to pay for or towards that which has been provided. He is not sure even whether such charges may not be illegal altogether under the Act. Further, he dwelt on the difficulties that may arise in treating patients in adjacent beds, one of whom pays five shillings a week out of his compensation, while the other pays nothing: questions of privilege may thus easily be raised. In the Leicester Infirmary last year not more than 80 out of 3,000 in-patients were in receipt of compensation, and the total received from them under a system of contribution from this source would not have been more than £120. In the casualty department 15 per cent. of the out-patients were in receipt of compensation, but the average cost of treating these cases, which alone could be recovered, was but two shillings. He suggested that the better way is to ask the working classes to contribute regularly to hospitals during health, instead of waiting until they are under treatment, and then demanding part of their compensation money.

Dr. J. A. MacDonald said that hard and fast rules should not be made, but that cases should be considered on their merits. He referred to the question of clubs, and said that a man receiving compensation from his employers and also sick pay from two or three benefit societies may very well be called upon to pay something to the cost of his treatment in hospital.

Questions were then asked as to the provisions of the Act and of insurance policies under the Act with reference to certain clauses in them that sums up to £10 may be allowed for necessary medical treatment, and views were expressed that hospitals should benefit by such sums. Finally an amendment was proposed by Dr. F. Fowler, of Bournemouth, to add to the motion "whom the Hospital Board shall consider unable to pay for medical attendance." This was accepted by Mr. Johnson, and carried, as was also the amended motion.

#### Payments for Drugs.

The Hon. Sydney Holland, Chairman of the London Hospital, then moved "That where they can afford to do so hospital patients ought to be asked to pay for the food, medicines, and bandages supplied to them." He dwelt on the increasing difficulty of raising funds for hospitals, a difficulty which in his view will ultimately bring about State aid. But one of the mistakes of the present system is that whether patients can pay or not, they are never asked to pay for what the hospital supplies. He read a series of axioms on which to base the refutation of the arguments against his motion. To give everybody everything for nothing, whether they are able to pay for it or not, is to pauperise the people. Last year payments for medicines, etc., were made by 157,000 patients at the London Hospital without any grumbling, and 4,500 who at first pleaded inability were afterwards compelled to contribute. The result was £2,000 for the coffers of the hospital. One patient out of every seven was excused payment on the score of destitution, but at the Poplar Hospital only one in 90 was thus unable to contribute.

The objection of the medical staff is that payment for medicines reduces them to the position of sixpenny doctors, and that it can never be made clear that the payments are for medicine and not for treatment. This objection is disposed of by the great care which is taken to impress upon all patients the true state of things, and by the fact, well-known among the out-patients, that if no medicines or bandages are ordered the threepence collected is returned. There is, at the same time, no doubt that a very few patients do believe that threepence represents the value of the advice and treatment they receive, but this number is so small that it need not be considered. General practitioners are afraid that this system of small charges at hospitals will result in some patients resorting there who would not accept charity if it was absolutely free. Mr. Holland thinks that this fear has not been, and will not be, realised in practice.

Lieut-Col. Montefiore, of the Metropolitan Hospital, said that any man who can afford to pay for bandages and so forth, ought to belong to a sick club and so benefit the medical profession instead of hospital funds. He does believe that many poor people think they are paying for advice as well as medicine, and opposed the resolution entirely.

Mr. J. Dyson, of Crewkerne Hospital, asked if these principles were held to apply to small and cottage hospitals as well as to large ones. Mr. Holland replied that what was sought was to extend the system common in cottage hospitals to the larger institutions also.

Mr. Walter Carnt, of the Manchester Infirmary, explained the system of voluntary payments, which produces £1,100 annually for that institution. Out-patients are charged only when they have been sent by a practitioner, and some such cases pay as much as 1s. 6d. per attendance. As there is a wage limit of 21s. a week for the out-patient department, except for cases sent for special treatment by medical men, probably not many of those now treated free can afford to pay for medicine.

Rev. W. Blissard, Kent and Canterbury Hospital, speaking as a manager of a small provincial hospital, thinks the effect of charging for board will be to increase the number of patients and to injure the medical profession. The effect on the charitable public must also be considered, for they subscribe to hospitals in order to benefit their very poor neighbours, but they may not be so much inclined to do so for those who are much less in need of assistance.

Dr. G. A. Heron, City of London Hospital for Diseases of the Chest, agreed with Mr. Holland that the ultimate fate of the hospitals is State maintenance, but protested very strongly against many other of his statements and opposed the motion. He regards the almoner as one of the greatest delusions of the present day, and gave instances occurring within his own knowledge of gross hospital abuse by well-to-do people. No man who can afford to pay a general practitioner should be permitted to go into a hospital. He hopes that Mr. Holland will come to see that his proposed system of payments strikes at the root of all true charity.

Mr. Holland, in reply, said that charging patients for food, medicine, or bandages as a fact neither increases nor decreases the number of patients, supporting this by statistics. Not only is there no effect on the numbers applying, there is also no effect on the hospital income from subscriptions. The class that hospitals exist to benefit is the class just above the wholly destitute, for whom Poor-law infirmaries are provided. He quoted a case from his own knowledge of a clerk earning £250 a year who was charged in all £160 for an operation by a surgeon in a nursing home.

The motion was carried by a fair majority.

### The Value of Almoners.

The next motion on the list was proposed by Lieut.-Col. Montefiore, and aroused much less controversy than the previous ones. It was "That in the opinion of this Conference, the appointment of trained hospital almoners constitutes one of the best methods for checking the misuse of hospitals, and for rendering more effective the economic value of an out-patient department." The proposer said that the third of the "Model Principles" requires that some means of investigation into the circumstances of the applicants for relief, by means of an almoner or other agent, shall be employed in all medical charities. The best way of securing this end is by the appointment of trained almoners. Some nine or ten of the best-known hospitals in London have already followed the example set some years ago by the Royal Free Hospital, and others are contemplating doing so, by the appointment of almoners. He explained in detail the province and duties of an almoner, and gave instances of the way in which deserving cases are brought to notice as well as of that in which imposture is prevented. Tact, common sense, patience, and devotion are the qualities necessary; but training and experience are also essential. The almoner must be thoroughly acquainted with the lives of the poor, and should work for some months in the local office of the Charity Organisation Society, and after that for some time under a trained and experienced almoner at a hospital.

Hon. S. Holland said there is general agreement that almoners are required, but he puts the percentage of ineligibles that apply at not more than two or three per cent. There is, as a fact, much less hospital abuse than is often thought and stated to exist. He thinks Col. Montefiore's list of the almoners' duties rather too comprehensive, as, for instance, in the matter of seeing that the physician's orders are duly carried out.

Dr. Norman Moore, St. Bartholomew's Hospital, corroborated Mr. Holland as to the satisfactorily small extent of hospital abuse. He also referred to the investigation of real cases of distress which are suitable for relief out of the Hospital Samaritan Fund.

Mr. Dodson, Sussex County Hospital, also thinks the system works well there, half the cost of the almoner's office being borne by the Charity Organisation Society, though the Hospital Board have full power of dismissal, etc. At that hospital the almoner distinguishes three classes of patient; those who cannot pay at all for medical treatment, those who can pay for ordinary but not for special treatment, and those who are ineligible altogether. The second class are allowed to receive only special treatment. The indirect effect of the appointment of an almoner is much greater than the direct; that is, many more ineligibles are deterred from attempting to obtain treatment than are actually refused it. He associated himself with the previous speakers as to the value of an almoner's office in the discovery of deserving cases.

The motion was carried with but one or two dissentients.

It was resolved, *nem. con.*, on the motion of Mr. H. A. Harben, St. Mary's Hospital, "That the use of the out-patient departments of hospitals by persons able to pay for their own treatment can be most effectually prevented by a close co-operation between hospitals, on the one hand, and provident dispensaries and similar institutions and local medical practitioners, on the other."

### Cottage Hospital Charges.

The Conference was resumed on Thursday morning, Mr. Charles Lupton again presiding.

Colonel J. G. Curtis (Deal and Walmer Victoria Hospital) moved: "That some definite steps should be taken to ascertain the ability or contrary of every patient of a cottage hospital to pay for his maintenance."

So far as he can discover, there is no general scheme of charges among cottage hospitals. Some leave the matter to the gratitude of patients, some have a fixed scale of charges, some charge according to the rent of the house in which a patient lives, and others according to the wages he earns. In some hospitals there is a system of subscribers' letters under which patients are admitted at reduced charges. It is desirable that a general system should be adopted by which the hospital should be able to collect the money due and yet to prevent the pauperisation of poor patients.

Mr. C. Grant (Beckenham) said the hospital he is associated with makes a minimum charge of 3s. 6d. per week. They have an out-patients' department, and make a charge of 1s. for minor operations such as tooth extractions. They had no subscribers' letters at first, but when the hospital was enlarged this system was introduced. He has not found that there is any increase of subscriptions from that cause, and a large proportion of the subscribers are now in favour of doing without such letters.

Dr. Dickson (Marlow) said it would be a mistake to run cottage hospitals upon hard and fast lines. The great point is to get patients back to their work as quickly as possible.

Dr. Pope (Leicester) contended that it is unfair to the local practitioners for a cottage hospital to encourage out-patients, to extract teeth, and to dispense medicines.

Dr. James Hamilton (member of Council) said the hospital with which he is connected has recently abolished subscribers' letters because they are found to be of no benefit to the public. He thought a minimum charge of 3s. 6d. might be adopted.

The resolution was carried.

### Uniform Hospital Accounts.

Dr. E. L. Fox moved: "That in the opinion of this Conference the uniform system of accounts recommended by the King Edward's Hospital Fund should be adopted as far as possible at all hospitals."

It is said that doctors do not know anything about finance. He does not dispute it. It is also said that doctors do not know how to make money. He does not dispute that either. (Laughter.) It would, however, be a matter of great convenience to doctors and to the public if this scheme were adopted. It was suggested by Sir Henry Burdett, and its adoption means greater economy, less trouble to the authorities of the hospital, and the putting down of waste and extravagance. It means that certain averages can be worked out as to the cost of each bed. This is not an infallible test, but it is a great help. If every hospital were to do this, and some means were taken to give publicity to the figures it would be of immense assistance.

Mr. Lewis (Maidstone) said he cannot see how such figures will assist. If, on the other hands, some well-reasoned figure as to cost can be arrived at it might be of some service. He suggested as an amendment that the committee should be instructed to bring forward a scheme for estimating the expenditure of the out-patient department.

Mr. A. F. Graves (Sussex Eye Hospital) was at one time opposed to the King Edward scheme, because he thought it might greatly increase his work, but he finds upon examination that the system can be so simplified as to make it quite easy to give all the practical and useful information required.

Dr. Fox asked leave to withdraw the resolution and to substitute the following: That the Conference instructs the Committee to consider how a uniform system of accounts can be made applicable to the requirements of provincial hospitals, and to make recommendations to the next conference.

The resolution was carried.

### County Guilds and the League of Mercy.

Mr. R. H. Kinsey (Bedford County Hospital) moved a resolution approving of the scheme of the Bedfordshire Hospital Guild as a means of increasing local interest in county hospitals. He desired to bring the scheme of the Bedford Hospital before the Conference because he believes that if it were followed it would help to solve some of the questions debated on the previous day. The scheme involves the organisation of a guild, the purpose of which is to obtain a large number of small subscriptions, to increase the utility of the hospital by doing supplementary charitable work, and to utilise the assistance of a large number of charitable ladies and gentlemen. The main idea of the scheme is to interest the people of the district in the hospital and to tap a source of revenue which has hitherto been left untouched, namely, the small subscription.

Lord Ampthill (Chairman of the Bedford County Hospital) said there is absolutely nothing new in the scheme. It is simply a measure for co-ordinating and systematising existing efforts so as to prevent overlapping and to secure that there should be an equal distribution throughout the whole area in question of all the public-spirited efforts which are already made. It is, in fact, a decentralisation of effort and a concentration of control.

Dr. E. L. Fox moved the following amendment: "That in the opinion of this Conference the time has come when the consideration of the King should be called to the condition of provincial hospitals, and sanction be asked for individual counties to start a King Edward's Hospital Fund of their own."

The Chairman ruled the amendment out of order.

A member of the Conference moved as an amendment that the words "has heard with interest" be substituted for the word "approves."

The amendment was carried and the resolution was adopted in the following form: "That this Conference has heard with interest of the scheme of the Bedfordshire Hospital Guild, as a means of increasing local interest in the County Hospital and extending its usefulness by promoting more intelligent efforts for the prevention of disease and for the better after-care of convalescents.

### Notification of Tuberculosis.

At the afternoon session the Rev. W. Blissard (Kent and Canterbury Hospital) moved "That tuberculosis should be included among notifiable diseases with a view to the isolation of such patients in publicly provided hospitals." He said it needs no words to impress that assembly with the seriousness of this disease. Hospital accommodation for it is altogether inadequate and private charity cannot be expected adequately to meet the need. As to tuberculosis, it is now pretty well accepted that heredity only affects it in the limited sense that children of tuberculous parents may be more susceptible to bad environment. What must be looked to is the environment which is producing so large an increase in tuberculosis. It may be said that the spread of consumption is one of the pathological symptoms of national prosperity. Tuberculosis is a consequence of the activities which are building up the wealth, the luxury, and the prosperity of the nation, and it is from that point of view that

ARMY MEDICAL LIBRARY

he recommends public provision for tuberculous patients. Of course the expense would be very great, but unquestionably the cost of leaving things as they are must be far greater, and apart from the financial aspect they are wasting the physical energies of the nation.

Dr. Henry Davy, President of the British Medical Association, moved as an amendment that the consideration of the propriety of the notification of tuberculosis be postponed, and that the Government be asked to appoint a Royal Commission to consider the best way to deal with tuberculosis. He is entirely sympathetic, he said, to Mr. Blissard's motion; he thinks it most important that tuberculosis should be included among notifiable diseases; but that is far from being a simple matter. Notification alone is no good whatever. No doubt one great difficulty in getting the Government to appoint a Commission is the number of inquiries that have already been held. Dr. Bulstrode published the result of his investigations, and his opinion seems to be that the disease will die out as sanitation improves. He (the speaker) acknowledges the immense amount of information Dr. Bulstrode has acquired, but, while there are 60,000 deaths each year, he is not content to leave the subject alone.

Dr. Heron seconded the amendment. He finds no fault at all with the speech of the mover of the resolution, and he heartily concurs in his conclusions. But if a man is told he must be notified as a victim of an infectious disease, while he is under the consequences of that notification the country must take care of him lest he should infect others, and also take care of his wife and family. If for the national welfare a patient is isolated, he cannot be told to look to charity to avert the dire consequences of that step. Tuberculosis is in these days pre-eminently a disease of the poor; it is remarkable how seldom, comparatively speaking, though still too often, well-to-do people die of it. The cost must, therefore, be a matter of millions, probably.

After further discussion the amendment was put and carried *nem. con.*, the mover of the original resolution expressing his acceptance and welcome of the proposal for a Royal Commission.

The new committee was appointed with instructions to organise a Conference in 1909, the 16 members elected by the Conference being as follows :—Mr. Walter Bailey, Mr. R. Ball-Dodson, Sir Henry Burdett, Mr. W. G. Carnt, Colonel Curtis, Mr. W. A. Dinwiddie, the Hon. Sydney Holland, Mr. Gosnell, Mr. H. Harben, Mr. Jewson, Sir Riley Lord, Mr. Charles Lupton, Mr. H. Manfield, M.P., Mr. H. W. Mason, Colonel Montefiore, and Mr. Leonard Rea.

